# Targeting protein synthesis pathways in MYC-amplified medulloblastoma

**DOI:** 10.1007/s12672-025-01761-7

**Published:** 2025-01-08

**Authors:** Devendra Kumar, Ranjana Kanchan, Nagendra K. Chaturvedi

**Affiliations:** 1 Department of Pediatrics, Division of Hematology/Oncology, University of Nebraska Medical Center, Omaha, NE 986395 USA; 2 Department of Biochemistry and Molecular Biology, University of Nebraska Medical Center, Omaha, NE USA; 3 Child Health Research Institute, University of Nebraska Medical Center, Omaha, NE USA; 4 Fred & Pamela Buffett Cancer Center, University of Nebraska Medical Center, Omaha, NE USA

**Keywords:** Brain cancer, Medulloblastoma, MYC, Protein synthesis, MTOR pathway

## Abstract

MYC is one of the most deregulated oncogenic transcription factors in human cancers. MYC amplification/or overexpression is most common in Group 3 medulloblastoma and is positively associated with poor prognosis. MYC is known to regulate the transcription of major components of protein synthesis (translation) machinery, leading to promoted rates of protein synthesis and tumorigenesis. MTOR signaling-driven deregulated protein synthesis is widespread in various cancers, including medulloblastoma, which can promote the stabilization of MYC. Indeed, our previous studies demonstrate that the key components of protein synthesis machinery, including mTOR signaling and MYC targets, are overexpressed and activated in MYC-amplified medulloblastoma, confirming MYC-dependent addiction of enhanced protein synthesis in medulloblastoma. Further, targeting this enhanced protein synthesis pathway with combined inhibition of MYC transcription and mTOR translation by small-molecule inhibitors, demonstrates preclinical synergistic anti-tumor potential against MYC-driven medulloblastoma in vitro and in vivo. Thus, inhibiting enhanced protein synthesis by targeting the MYC indirectly and mTOR pathways together may present a highly appropriate strategy for treating MYC-driven medulloblastoma and other MYC-addicted cancers. Evidence strongly proposes that MYC/mTOR-driven tumorigenic signaling can predominantly control the translational machinery to elicit cooperative effects on increased cell proliferation, cell cycle progression, and genome dysregulation as a mechanism of cancer initiation. Several small molecule inhibitors of targeting MYC indirectly and mTOR signaling have been developed and used clinically with immunosuppressants and chemotherapy in multiple cancers. Only a few of them have been investigated as treatments for medulloblastoma and other pediatric tumors. This review explores concurrent targeting of MYC and mTOR signaling against MYC-driven medulloblastoma. Based on existing evidence, targeting of MYC and mTOR pathways together produces functional synergy that could be the basis for effective therapies against medulloblastoma.

## Introduction

Medulloblastoma is the most common pediatric brain tumor of neuroectodermal cerebellar origin, accounting for approximately 20% of all childhood brain tumors and over 60% of embryonal brain tumors. Approximately one third of children with medulloblastoma succumb to the tumor even after receiving standard surgery, chemotherapy, or radiation treatments. Moreover, because of such treatments, surviving patients suffer severe long-term side effects including neurocognitive defects [1, 2]. Extensive genetic, epigenetic, and transcriptomic analyses have identified medulloblastoma as a heterogenous disease with four major molecular subgroups, namely wingless (WNT pathway-activated), sonic-hedgehog (SHH pathway-activated), Group 3 and Group 4 [3–5]. Of these, Group 3 medulloblastoma represents the most aggressive subgroup (with < 60% overall survival) which often exhibits MYC amplification or overexpression (17–20% of cases), metastasis (40–50% of cases), and treatment resistance [6–8]. Thus, there is an urgent and unmet need to develop new targeted therapies for treating such medulloblastoma while acquiring limited toxicities.

Dysregulation of protein synthesis caused by abnormal activation of oncogenic signaling pathways has arisen as a critical mechanism for cancer progression and therapy resistance [9, 10]. Deregulation of protein synthesis is driven by uncontrolled expression of MYC, a transcription factor that is often deregulated by chromosomal aberration, retroviral insertion, activation of super-enhancer with *MYC* gene, or mutation of upstream signaling pathways in various cancers including medulloblastoma [11]. Studies have shown that the oncogenic effect of MYC is due to increased protein synthesis, fueling increased cell size and proliferation. The dramatic increase in cell protein synthesis that occurs after MYC activation stems from transcriptional modulation of multiple protein-synthesis components, including mRNA translational factors and ribosomal biogenesis [12–14]. The mRNA translation is also enhanced by the activation of mammalian targeted rapamycin (mTOR) kinase-dependent phosphorylation of the tumor suppressor eukaryotic translation initiation factor 4E (eIF4E) binding protein (4EBP1) [15]. MYC stimulates the hyperactivation of eIF4E to drive tumorigenesis, and mTOR stabilizes MYC levels by inducing MYC translations [16, 17]. MTOR is one of the major pathways known to be activated during medulloblastoma progression. MTOR signaling coordinates organismal development and homeostasis, encompassing lipid and protein synthesis that govern the cell cycle and cellular metabolism [18–20].

Biologically targeted therapies are better tolerated than conventional therapies and have extended patient survival with minimal or no toxicity [21]. MYC is a highly warranted therapeutic target due to its broad role in cancer development, its overexpression in variety of cancers (> 50% of all cancers), and its association with therapy resistance and poor prognosis [22]. Currently, no effective small-molecule therapeutic agents are available to target MYC protein because of a complex protein structure, non-enzymatic nature and short half-life. Drug discovery approaches attempted at blocking MYC heterodimerization with MAX or its binding to DNA elements in the target gene promoters, to date, largely failed [22, 23]. Although targeting MYC with alternative or indirect strategies such as blocking its upstream or downstream signaling have been promising, MYC remains challenging to target due to its wide roles and the number of tumorigenic pathways modulated by it. Aggressive tumors are often more resistance to conventional treatments such as radiation and chemotherapy [24]. The activation of mTOR pathway has been shown to be involved in such resistance in cancers, including medulloblastoma. This review updates recent findings on the crosstalk between MYC and mTOR and targeted therapies that inhibit both MYC and mTOR along with other treatment modalities that hold potential to treat the Group 3 MYC-amplified medulloblastoma at the translational level.

## Tumorigenic roles of MYC-induced protein synthesis

**Fig. 1:**
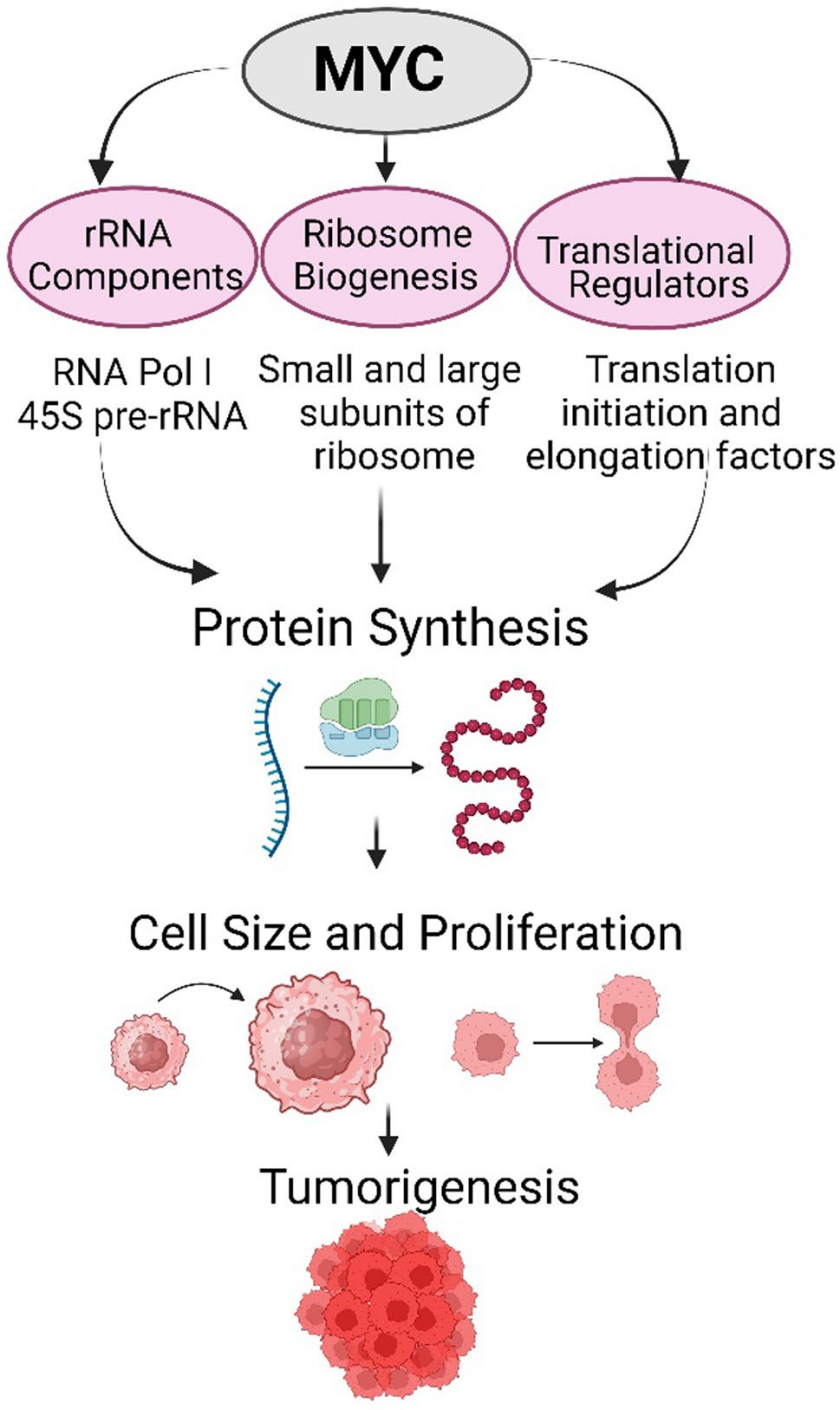
Tumorigenic effect of MYC by regulating the transcription and translation machinery. MYC promotes transcription of several components of protein synthesis machinery as indicated thereby increases cell mass and proliferation in cancer cells

The MYC transcription factor is one of the most activated oncogenes in human cancer. Particularly, MYC overexpression correlates with poor clinical outcomes and worse survival in a wide range of cancers including medulloblastoma [25]. When MYC is activated, it can direct uncontrolled cell proliferation, leading to tumorigenesis (Fig. 1). Deregulation in multiple steps of protein synthesis control is an emerging mechanism for cancer progression. MYC directly increases protein synthesis rates by controlling the transcription of protein synthesis machinery components, including mRNA translation, ribosome biogenesis (ribosomal small and large subunit proteins) components and translation initiation/elongation factors [26–29]. Increased production of ribosomal proteins can boost the capacity of the cells for protein synthesis, possibly fueling the instant growth of cancer cells. MYC could control several translation factors involved in protein synthesis and confirm the expression changes associated with MYC oncogenic function [30–34]. In particular, the strong upregulation of genes encoding RNA polymerase I (Pol I) complex, which is responsible for transcription of the 45S pre-rRNA encoding genes (rDNA), is a crucial mediator of MYC-enhanced gene expression [35]. rDNA is a critical rate-limiting step for ribosomal biogenesis and could be targeted by small molecular inhibitors. A recent study has shown ribosomal biogenesis can be suppressed by inhibiting the rDNA using a small molecule CX-5461, which has the capacity to control or kill the MYC-driven cancer cells. This inhibitor is currently in a Phase-I clinical trial [36, 37]. Thus, controlling the ribosomal biogenesis at multiple points offers a possible strategy to treat MYC-driven medulloblastoma [38]. Interestingly, in our recent study, we find that the key components of protein synthesis machinery, including mTOR signaling and MYC targets, are overexpressed and activated in MYC-amplified medulloblastoma cell line models [39], confirming the role(s) of MYC-induced protein synthesis in medulloblastoma tomorigenesis.

MYC-dependent increase in protein translation also controls the genome variability. The initiation of cap-dependent translation usually slows down in the stage of mitosis. However, Internal ribosome entry site (IRES) dependent translation promotes the expression of critical cytokinesis regulators involved in cell cycle progression by restricting the switch between cap and IRES-dependent translation [14, 40, 41]. MYC itself has IRES elements in its UTR [42]. Because of MYC hyperactivation, the failure of cytokinesis was accompanied by an excess number of centromeres, restored in conditions of normal protein synthesis [14].

MYC activation can increase protein mass by directly controlling the translation of specific mRNAs. An understanding of this mechanism came from the observation that MYC leads to an increase in the levels of several cyclins, thereby affecting the activities of cyclin-dependent kinases (CDKs), which are required in in G1 transition of cell cycle and cell division. CDK levels are abundantly increased in response to MYC overexpression, despite no change in their RNA levels [43, 44]. MYC was shown to enhance the translation of individual mRNA by promoting methylation on the 5’ region of the mRNA (mRNA 5’ capping), which is necessary for binding the translation factors to the mRNA [45, 46]. 5’ mRNA capping is essential for mRNA stability, as uncapped RNA degrades rapidly. MYC induces mRNA cap methylation, revealing that it can be an important mechanism to stabilize mRNA translation for some genes [43]. However, MYC has no direct role in mRNA capping; instead it can directly regulate transcription of genes that are involved in mRNA capping. For example, MYC promotes transcription of TFIIH (basal transcription factor) that phosphorylates RNA Poll II [47]. One of the subunits of TFIIH is CDK7, which has kinase and cyclin-dependent activating kinase (CAK) activities that phosphorylate the C-terminal domain of RNA Pol II. MYC also controls the expression of CDK7 and other CDKs [48]. MYC forms MAX-independent complex with TFIIIB and control gene transcription, including genes involved in the Pol III transcription machinery and small RNAs [49, 50].

Additionally, MYC and E2F1 (a transcription factor) can directly promote methylation of mRNA CAP structure through RNA guanosine-7-methyltransferase (RNMT), a modification essential for CAP bonding to eIF4E and recruitment of 40S ribosomal subunit that lead to CAP-dependent translation initiation [44]. MYC’s role in upregulating rRNA transcription also indirectly affects translation initiation. Ribosomal promoters L13, L19, L22, L27A, and S6 are also confirmed high-affinity MYC binding sites. MYC’s promotion of rRNA gene transcription leads to increased ribosome production, supporting translation initiation by providing more ribosomes for protein synthesis [51]. It frequently boosts the transcription of growth-promoting genes, some of which encode translation initiation factors, including eIF4E, which is implicated in translation initiation and required for CAP-dependent translation [51]. The translation initiation factors eIF4A and eIF5A, including eIF4E, contain high-affinity MYC-binding sites. Recently, researchers developed a constitutive active 4EBP1 inhibitor to target eIF4E [52]. The 4EBP1 inhibitor antagonizes eIF4E by signal transduction pathways that phosphorylate and inactivate of 4EBP1, suggesting the potential importance of eIF4E as a MYC regulatory target in cancer. One of the most surprising discoveries over the last several years is that, contradictory to preceding acceptance, eIF4E expression is not a controlling factor for overall protein translation. Even if the eIF4E level is reduced by 50%, it still does not impact normal development and translation globally; however, a reduction in eIF4E expression would be expected to suppress oncogenic transformation [53]. FDA-approved antiviral drug ribavirin has been shown to suppress eIF4E in cancer [54]. Ribavirin could be a valuable addition for MYC-amplified medulloblastoma targeted to eIF4E. Decisively, eIF4E overexpression alone is sufficient to act as driving oncogenic events, and overexpression of eIF4E through inhibition of 4EBP1 is required for mTOR-dependent tumorigenesis [17, 19], which creates a unique window of prospect for pharmacological intervention. LY2275796, which blocks the expression of eIF4E, was in a Phase I clinical trial (NCT00903708) that sought an appropriate dose of LY2275796 in patients with advanced tumors [55]. Another translation initiation factor, eIF4A (a helicase), is a crucial member of the eIF4F complex that regulates pro-cancerous signaling. eIF4A liberates secondary structures in the 5’ untranslated region (UTR) to help scan the 43S complex to recognize the start codon. Hence, it is believed to be inappropriate for translating mRNAs with complex 5’ UTR. eIF4A has two paralogs with 90% homology at the amino acid levels (eIF4A1 and eIF4A2). eIF4A1, a crucial transcriptional target of MYC [56], is frequently overexpressed in various malignancies and was shown to facilitate the translation of numerous oncogenes [57]. A recent study showed that decreased eIF4A1 levels suppress lymphomagenesis in murine MYC-driven lymphoma [58], suggesting that eIF4A1 is a viable target for cancer therapy. The eIF4A inhibitor, eFT226 (Zotatifin), is already in Phase I/II clinical trial (NCT04092673) to treat solid tumor malignancies. However, the impact of translation elongation factors in the cancer perspective is poorly understood. One of the elongation factors involved in translation is eIF5A. It was formerly known as an initiation factor; however, some studies show its main role in translation elongation. The eIF5A was classified into two isoforms, eIF5A1 and eIF5A2, based on posttranslational modification. eIF5A1 is universally found in cells of most tissues, whereas eIF5A2 is exclusively found in the testis and brain [59] and primarily expressed in cancerous cells [60, 61]. Recently, a study showed that eIF5A regulates the selection of MYC-mRNA start codon in cancer cells [62]. Similarly, eIF5A may more generally regulate selective translation of oncogene tripeptide (Met-Phe-Phe) or proline stretches, which need eIF5A movement to avert ribosome stalling [63]. Early research on the function of eIF5A as a translational regulator in cancer suggests that it may be a promising therapeutic target.

By regulating ribosome biogenesis and translation, MYC can exert coordinated control of cellular protein production, leading to cell growth and cell division. Overall, findings suggest that deregulation in protein synthesis downstream of MYC can have an immediate and profound effect by causing additional genetic lesions that cooperate with MYC hyperactivation in cancers including medulloblastoma.

## Co-operation and crosstalk between MYC and mTOR signaling

Protein synthesis is not only enriched by MYC-regulated transcription but also by the activation of mTOR kinase at the translation level. MTOR signaling itself is another key regulator of protein synthesis which is frequently deregulated in various cancers, including MYC-addicted cancers and medulloblastoma [64]. MTOR has two distinct protein complexes, mTORC1 and mTORC2. MTORC1 is a primary regulator of cell growth and metabolism. It associates with raptor, mLST8, PRAS40, and DEPTOR and integrates various signals, including nutrient availability and growth factors that control processes like protein synthesis. MTORC2 is associated with mLST8, mSn1, Protor1/2 and DEPTOR. It primarily regulates cell survival, proliferation, and cytoskeletal organization and is insensitive to rapamycin. The distinct functions of these complexes and their integration with other signaling pathways make them central players in regulating cell behavior and physiology [65].

**Fig. 2:**
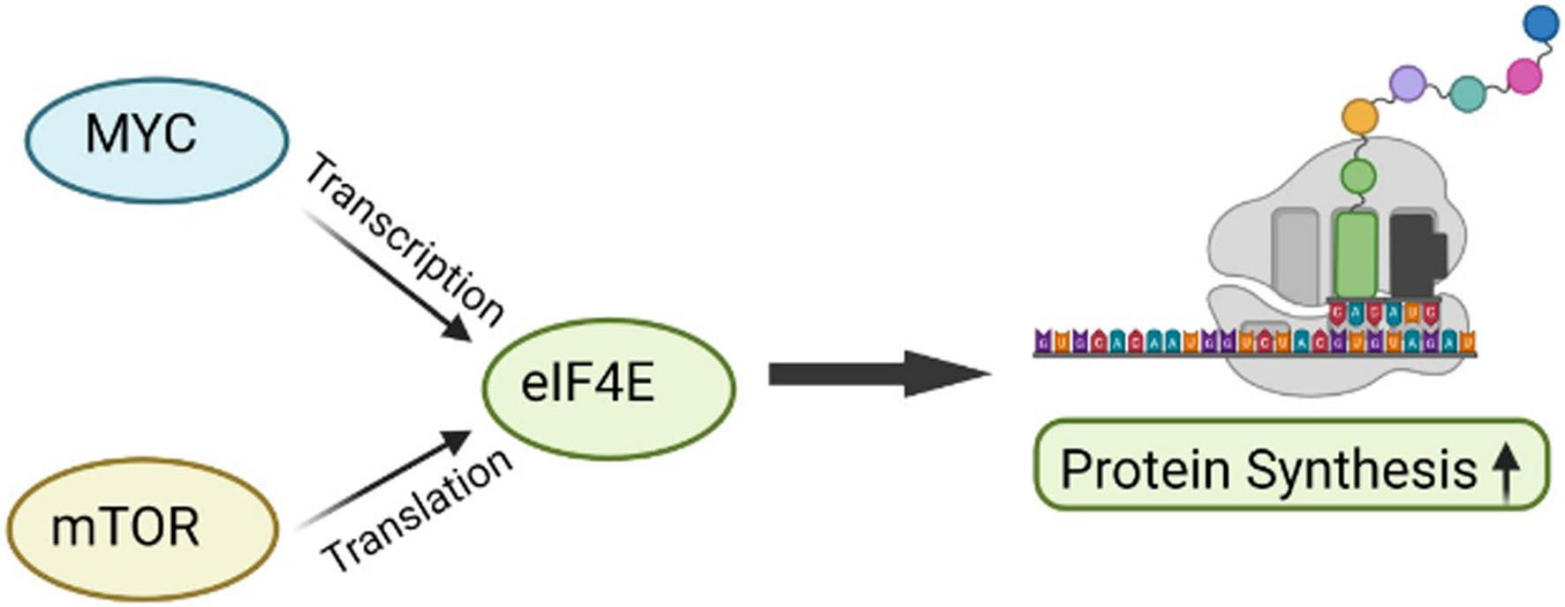
Interaction and cooperative crosstalk between MYC and mTOR to enhance the protein synthesis in cancer progression. This figure is showing both MYC (at transcription) and mTOR (at translation) connects at the primary iniation translation site eIF4E to enhance global protein synthesis in cancer cells

MTOR controls protein synthesis by phosphorylating the tumor suppressor 4EBP1 and ribosomal protein p70S6 kinase (S6K). MTOR-dependent phosphorylation of 4EBP1 blocks its ability to negatively regulate the translation initiation factor eIF4E, thus promoting eIF4E’s ability to initiate protein translation (Fig. 2) [19]. Importantly, it has been established that MYC stimulates hyperactivation of eIF4E to drive tumorigenesis. Also, MYC stimulates mTOR activity indirectly by promoting the expression of growth-promoting factors that activate the mTOR signaling pathway. On the other hand, it has been shown that mTOR also stabilizes the MYC protein concentration by inducing more MYC exression. Together, these studies support the idea that crosstalk between MYC- and mTOR-dependent mechanisms of translation reprogramming leads to enhanced protein synthesis, which is required to sustain the oncogenic drive. Therefore, the MYC/mTOR axis is an attractive therapeutic target in MYC-driven cancers that are addicted to enhanced protein synthesis.

The interactions between MYC and mTOR signaling have been well studied in the lymphoid malignant microenvironment. This phenomenon is now emerging in other cancers as well. Interestingly, studies by us and others have shown that mTOR signaling is overactivated in Group 3 (MYC-amplified) medulloblastoma, suggesting association between MYC and mTOR in medulloblastoma. Particularly, MYC and mTOR cooperatively control the primary protein synthesis/translation step (4EBP1/eIF4E) at the transcription and translation levels, respectively. These findings uncover an important link between MYC and mTOR-dependent protein synthesis/translation, which together lead to enhanced tumorigenesis. Cooperation between these two pathways may dysregulate translation globally and promote the pathology of MYC-dependent cancers, including medulloblastoma. Future studies addressing the molecular mechanism(s) for MYC/mTOR interaction may provide important insights into how this interaction is regulated under normal and pathological cellular conditions.

Another major and immediate downstream effect of MYC activation is a dramatic increase in metabolism of the cells as it directly upregulates energy/ATP production rates through transcriptional and protein synthesis control to sustain the uncontrolled cancer cell proliferation. MYC’s effects on cellular metabolism include making the cell more reliant on nutrients and energy sources. This metabolic shift and rewiring provide the necessary building blocks for further activating mTOR signaling and mTOR-driven protein synthesis [66]. MTOR senses the availability of amino acids and integrates this information into the control of protein synthesis. Adequate amino acid availability is required for mTOR to initiate translation effectively [67]. This metabolic reprogramming associated with protein synthesis control could be another point of cooperative interaction or crosstalk between MYC and mTOR.

## Other associated pathways of protein synthesis

In addition to mTOR, there are other pathways associated with protein synthesis in various cancers. Other notable pathways are MNK and AMPK which are interconnected with mTOR signaling. Activation of these pathways can promote protein synthesis, cell growth and contributing to cancer progression. These pathways often crosstalk and cooperate to promote aberrant protein synthesis and tumor growth in cancer.

### MNK

Apart from mTOR, MAPK-interacting kinases (MNK1 and MNK2) perform a role in cancer cell proliferation by influencing the translation process. Following the discovery of eIF4E and its crucial function in protein translation, scientists recognized that it is serine phosphorylated by MNKs, part of the mitogen activated protein kinase pathway (MAPK), which controls various cellular activities, including cell growth and proliferation [68, 69]. This phosphorylation performed by either MNK1 or MNK2, is supposed to enhance the translation of a subset of mRNAs, many of which showed the significance of MNKs in tumorigenesis [70, 71]. In the context of cancer, MNKs are involved in the phosphorylation of eIF4E [72]. The phosphorylation of eIF4E by MNKs enhances its ability to initiate the translation of specific mRNA molecules that encode proteins promoting cell cycle progression and survival [73]. MNK1 and MNK2 can be phosphorylated by extracellular signal regulated kinase (ERK) and p21 activated kinase 2 (PAK2) [74], while dephosphorylated, especially MNK1, by protein phosphatase 2 A (PP2A) [75]. Specific phosphorylation and dephosphorylation sites on MNKs were found to affect the binding to eIF4E and disturb the binding to eIF4G. Also, phosphorylated MNKs were recognized to bind with mTORC1 and allow the binding of TELO2 (cell cycle protein) to the complex, which triggers the mTORC1-dependent phosphorylation of downstream substrates [76]. A recent study demonstrated the relationship that mTORC1 phosphorylates MNK2 [77]. Targeting MNKs or the MAPK pathway presents possible therapeutic strategies to inhibit excessive cell growth in cancer. Since normal cell growth and development are not affected by MNKs inhibitors, MNKs are relevant targets in malignancy, due to their vitality in cancer cell signaling [78].

### AMPK

AMP-activated protein kinase (AMPK) is a key regulator of cellular energy metabolism, and it is known to influence the stability of MYC protein indirectly, thus linking cellular energy status to control of MYC-mediated cellular process [79]. Recently, a study has shown that deleting both catalytic subunits (*prkaa1 and prkaa2*) from AMPK inactivated the enzyme and decreased the expression of multiple genes related to protein translation, including mTORC1 in an SHH medulloblastoma model [80]. The downregulation of translation associated genes implied lowering mTORC1 activity, which was proven by finding reduced p4EBP1 levels as compared to a control tumor with intact AMPK catalytic subunits [80]. AMPK-associated metabolic adaptability may be crucial for brain tumor development [81, 82]. In SHH signaling AMPK has been shown to interact with GLI1 to suppress SHH activity [83]. Therapies that interrupt AMPK only transiently may be necessary for safety in pediatric patients [81, 84]. Understanding the mechanism(s) by which AMPK inhibition halts medulloblastoma cell proliferation and survival may allow the design of potential targeted therapies that exploit the role of AMPK in SHH-driven medulloblastoma and other cancers.

## Targeting protein synthesis as a cancer therapeutic approach

**Fig. 3:**
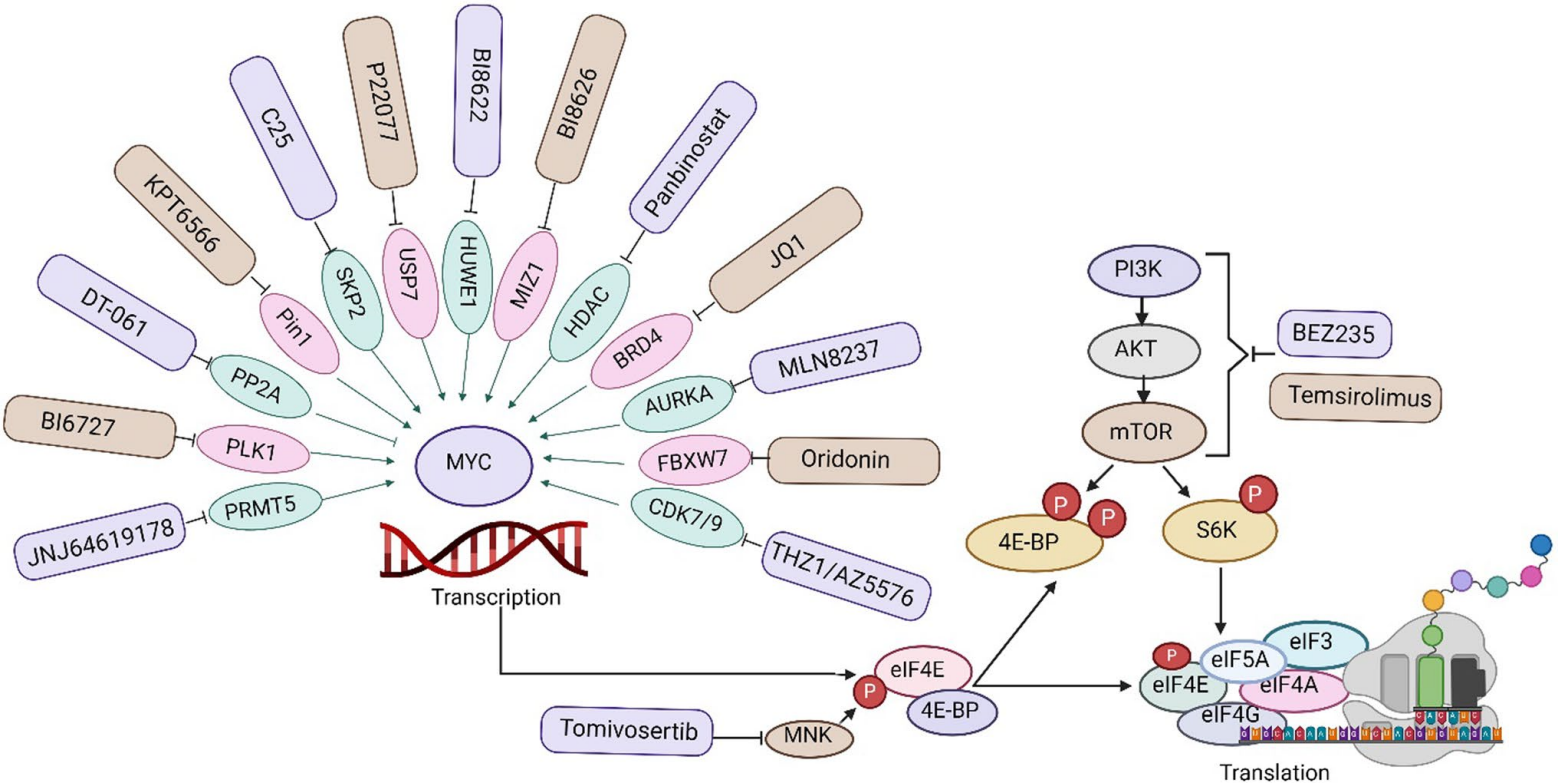
Possible combination strategies targeting MYC at transcription and protein translation levels

Understanding the crosstalk between MYC and mTOR is essential in cancer research and treatment. Targeting MYC and mTOR pathways may offer a more effective therapeutic approach in certain cancer-type, as it addresses multiple drivers for cancer growth and drug resistance. Inhibitor combination strategies that target mTOR signaling and MYC protein may be required to achieve complete blockade of the enhanced protein synthesis pathway (Fig. 3). Researchers are exploring combination therapy that inhibits both MYC and mTOR to improve treatment outcomes for MYC-driven cancer.

### Targeting MYC/MTOR

We review here the evidence that the MYC/mTOR axis may have attractive druggable targets for cancers addicted to enhanced protein synthesis [39]. Even though the MYC proteins themselves are undraggable, alternative strategies have recently been established that target MYC transcription and its regulated genes epigenetically by inhibiting bromodomain and extraterminal (BET)-containing proteins [22, 85]. BET proteins recognize acetylated lysines on euchromatin to facilitate transcription. In cancers, including medulloblastoma, MYC genes and their transcripts are specific targets for BET protein inhibitors [86]. Targeting BET proteins has been shown to effectively block cancer cells from eliciting a compensatory signaling response to PI3K pathway inhibitors; at least in some cases, this can restore sensitivity to therapy [87]. In ovarian cancer, it has been shown that resistance to BET inhibitors occurs through oncogenic kinome reprograming via the activation of receptor tyrosine kinases (RTKs) and downstream signaling of PI3K, AKT and ERK, which are compensatory pro-survival kinase networks [88]. Therefore, BET inhibitors may be thought of as rational combinatorial partners for reprogrammed compensatory signaling pathways such as PI3K-mTOR. The concept has been validated recently. Studies demonstrated that BET protein inhibitors and PI3K-mTOR ATP-active site inhibitors can facilitate therapeutic targeting of MYC and mTOR-dependent protein synthesis pathways, respectively [89, 90]. However, clinical experience with this approach is limited, and evidence obtained so far suggests that such agents have relatively poor anti-tumor efficacy individually. Recently, a combination of BET protein inhibitor JQ1 with a histone deacetylase inhibitor (panobinostat) synergistically induces anti-cancer effects in MYC-amplified medulloblastoma in vitro and in vivo [91]. Concurrent targeting of mTOR signaling and BET proteins may be necessary to achieve complete inhibition of the protein synthesis pathway. Our studies evaluated the anti-cancer potential of combined inhibition of MYC transcription and mTOR signaling in MYC-amplified medulloblastoma [39]. Combination therapy targeting MYC (by BET inhibition) and mTOR signaling proved efficacious against medulloblastoma [39]. In MYC-driven medullobalstoma cell lines, we observed that combined treatment with BET-MYC and mTOR signaling inhibitors at pharmacologically achievable doses, showed greater anti- medullobalstoma activity by downregulating the mTOR and MYC components. These results strongly support the rationale to further explore this therapeutic approach in MYC-driven medulloblastoma.

Resistance to mTOR inhibitors is common in cancer cells due to feedback activation of upstream PI3K kinase, furthering the rationale to combine inhibition of PI3K /mTOR with other targeted inhibitors to achieve a more durable blockade of mTOR signaling [92, 93]. Consequently, BEZ235, the dual inhibitor of PI3K/mTOR, was used to overcome this feedback activation and effectively target the mTOR-driven tumorigenicity [39]. BEZ235 has not yet been integrated into a clinical setting because of toxicity and lack of clinical efficacy in renal cell carcinoma patients [94]. Likewise, MYC and mTOR signaling activation has been demonstrated to synergize together in cancer biology, directing tumor deterioration and drug resistance in several malignancies, including medulloblastoma [95, 96]. Some targeted approaches may be explored in the context of MYC-amplified medulloblastoma and mTOR inhibitors (Fig. 3). We have illustrated the multiple pharmacological approaches to directly target mTOR at clinical level in Tables 1 and 2.

**Table 1:** Development of single or combination therapy approaches for targeting the mTOR signaling pathway to treat pediatric tumor

ClinicalTrials.gov identifier	Name of inhibitor	Patient groups	Conditions	Phase	Results/ Status
NCT01331135	Sirolimus with metronomic therapy	Children with recurrent or refractory solid and brain tumor	Ewing's Sarcoma, Osteosarcoma, Astrocytoma, Atypical Teratoid/Rhabdoid Tumor, Ependymoma, Germ Cell Tumor, Glioma, Medulloblastoma, Rhabdoid Tumor, Retinoblastoma, Clear Cell Sarcoma, Renal Cell Carcinoma, Wilms Tumor, Hepatoblastoma, Neuroblastoma, Rhabdomyosarcoma	Phase I	Well Tolerated/ Completed
NCT00187174	Everolimus	Pediatric Patients with Recurrent or Refractory Tumors	Tumors, Brain Tumors, Rhabdomyosarcoma Sarcoma, Soft Tissue	Phase I	Well Tolerated/ Completed
NCT00106353	Temsirolimus	Pediatric Patients with relapsed/ Refractory Tumors	High grade glioma, neuroblastoma, and Rhabdomyosarcoma	Phase I	Did not meet efficacy/ Completed
NCT01141244	Temsirolimus TemozolomideIrinotecan hydrochlorid	Pediatric Patients with relapsed/ Refractory Tumors	Unspecified Childhood Solid Tumor	Phase I	dose Tolerated/ Completed
NCT01049841	Temsirolimus Perifosine	Recurrent Pediatric Tumors	Solid Tumors	Phase I	toxicity Tolerated/ Completed
NCT01601184	Vismodigib temozolomide	Patients with meduloblastosma with an activation of SHH pathway	Histologically Confirmed medulloblastoma and Activation SHH Pathway	Phase I and II	Unclear/Terminated
NCT00776867	Perifosine	Recurrent Pediatric Solid Tumors	Solid Tumors	Phase I	toxicity Tolerated/ Completed
NCT02446431	Bevacizumab Cyclophosphamide Valproic acid Temsirlimus	Pediatric Patients with Solid Tumors at High Risk of Recurrence	Solid Tumor	Phase I	Recruiting
NCT04469530	Sirolimus Cyclophosphamide Etoposide Celecoxib	Children With High-Risk of Solid Tumors	Solid Tumor	Phase I	Recruiting
NCT03155620	Ensartinib Erdafitinib Larotrectinib Olaparib Palbociclib Samotolisib Selpercatinib Tazemetostat Tipifarnib Ulixertinib Vemurafenib	Pediatric Patients with Relapsed or Refractory Advanced Solid Tumors, Non-Hodgkin Lymphomas, or Histiocytic Disorders	Recurrent/Refractory Medulloblastoma, other solid tumors	Phase II	Recruiting
NCT00784914	Temsirolimus	Patients With Primary or Metastatic Brain Tumors	Solid Tumor	Phase I	Dose tolerated/ Completed
NCT03387020	Everolimus Ribociclib	Children With Recurrent or Refractory Malignant Brain Tumors	Solid Tumor	Phase I	Dose tolerated/ Completed
NCT03434262	A: ribociclib + gemcitabine B: ribociclib + trametinib C: ribociclib + sonidegib	Children and Young Adults with Recurrent Brain Tumors	Brain tumor, Medulloblastomas and tumors	Phase I	Active, Not Recruiting

**Table 2:** Development of single or combination therapy approaches for targeting mTOR signaling pathway to treat various cancers in adults

ClinicalTrials.gov identifier	Name of inhibitor	Conditions	Phase	Results/ Status
NCT02619864	AZD2014 + temozolomide	Glioblastoma Multiforme	Phase I	Dose tolerated/Completed
NCT02730923	AZD2014 + anastrozole	Solid Tumors but endometrial carcinosarcomas were excluded	Phase I and II	Dose tolerated/ Completed
NCT02208375	AZD2014 + olaparib and capivasertib (AZD5363)	Recurrent Endometrial, Triple Negative Breast, and Ovarian, Primary Peritoneal, or Fallopian Tube Cancer	Phase I and II	Active, not recruiting
NCT01548807	Everolimus with radiation therapy	Prostate Cancer Patients with Detectable PSA Following Prostatectomy	Phase I	Completed
NCT02831257	AZD2014	Neurofibromatosis 2 Patients with Progressive or Symptomatic Meningiomas	Phase II	Recruiting
NCT02397083	Everolimus + Levonorgestrel-Releasing Intrauterine System	Levonorgestrel-Releasing Intrauterine System with or without everolimus in Treating Patients with Atypical Hyperplasia or Stage IA Grade 1 Endometrial Cancer	Phase II	Recruiting
NCT02752204	AZD2014 + Rituximab	Relapsed or refractory Diffuse Large B Cell Lymphoma (DLBCL)	Phase II	Recruiting
NCT01899053	MLN0128 + MLN1117 (oral inhibitor of the PI3K (alpha) isoform)	Advanced Nonhematologic Malignancies	Phase I	Dose tolerated/ Completed
NCT02327169	MLN2480 MLN0128 Alisertib Paclitaxel Cetuximab Irinotecan	Advanced Nonhematologic Malignancies	Phase I	Dose tolerated/ Completed
NCT02193633	AZD2014 + Paclitaxel	Patients With Solid Tumors	Phase I	Dose tolerated/ Completed
NCT03648489	TAK228 + Paclitaxel	Advanced/Recurrent Epithelial Ovarian, Fallopian Tube or Primary Peritoneal Cancer (of Clear Cell, Endometrioid and High-Grade Serous Type, and Carcinosarcoma	Phase II	Completed
NCT00093080	AP23573/MK-8669 (Ridaforolimus)	advanced sarcoma	Phase II	Meet efficacy/Completed
NCT00331409	Everolimus + imatinib mesylate	Metastatic unresectable kidney cancer	Phase II	Completed
NCT01351350	MLN0128 paclitaxel trastuzumab	Advanced Solid Malignancies	Phase I	Dose tolerated/ Completed
NCT03730142	WXFL10030390	Advanced Solid tumors	Phase I	Pharmacokinetics, Dose toleration/ Completed
NCT02279758	Metformin	Well-differentiated Neuroendocrine Tumor	Phase II	Unknown status
NCT02684032	Gedatolisib Palbociclib Letrozole Fulvestrant	Breast Cancer	Phase I	Dose toleration/ Completed

Cyclin-dependent kinases (CDKs) are direct downstream targets of MYC which regulate cell cycle progression. Also, CDKs are involved in the phosphorylation events that can indirectly regulate MYC stability. Phosphorylation of MYC at specific sites can lead to its stabilization or degradation. For instance, phosphorylation at Serine 62 (Ser62) by CDK1 or CDK2 stabilizes MYC, whereas phosphorylation at Threonine 58 (Thr58) by GSK3β (which can be regulated by CDKs) marks MYC for degradation via the ubiquitin–proteasome pathway [97]. CDKs can interact with other regulatory proteins that influence MYC stability [98]. CDK inhibitors could be part of a treatment strategy for MYC-amplified medulloblastoma, although their precise role in controlling this specific cancer subtype is still an active area of research. CDK inhibitors, such as palbociclib or ribociclib, may be thought of in combination with mTOR-targeted therapy. Such a strategy may modulate the phosphorylation of MYC and interaction with other proteins, potentially diminishing the oncogenic effects. Recently, a combination of ribociclib with bet-bromodomain and PI3K/mTOR inhibitors was used for medulloblastoma treatment. The CDK inhibitor ribociclib inhibited MYC-driven and SHH medulloblastoma tumor progression models [99]. The combination of JQ1 and ribociclib potently repressed MYC expression and prevented the induction of its expression in group 3 MYC-amplified medulloblastoma cells [99]. BET and CDK inhibitors are often combined with other treatments, such as chemotherapy or targeted therapies, to address multiple aspects of cancer biology. Potentiation between inhibitors of BET and CDK was earlier shown in MYC-amplified group 3 medulloblastoma [100, 101]. A combination of CDK and mTOR inhibitors holds potential for controlling MYC-amplified medulloblastoma. PI3K/mTOR inhibitors have shown synergistic effects and advantages with BET and CDK inhibitors to treat group 3 and SHH medulloblastoma in preclinical tumor models [102–104]. The maximal advantage of combining CDK and PI3K/mTOR inhibitors might be achieved when combined with standard care [103]. The PI3K inhibitor, BKM-120, has shown a potently synergistic effect with histone deacetylase inhibitors to inhibit the tumor growth in vitro and in group 3 medulloblastoma models, identifying this as an effective combination therapy [105]. Some MYC-amplified medulloblastomas are associated with abnormal activation of SHH pathways. In specific cases, it may be deemed appropriate to target these pathways with inhibitors like vesmodegib or sonidegib in combination with mTOR inhibitors [106]. CDK and combinations can further control cancer growth by inhibiting MYC-amplified cell survival mechanisms and promoting apoptosis. While the exact mechanism of the combination therapy is still a subject of ongoing research, there are several ways in which these inhibitors may work together to target MYC-amplified medulloblastoma.

### Targeting MNK

MNK inhibitors are being explored as potential cancer treatments, particularly in cancers where the MAPK pathway is dysregulated. Some inhibitors are commercially available for laboratory work. Tomivosertib (eFT508), the most commonly used inhibitor, has the capacity to inhibit MNKs and p-eIF4E [107]. Now MNK inhibitors with improved pharmacokinetic properties, like ETC-206 and AUM001, are now available [108, 109]. Recently, the MNK1 inhibitor BAY1143269 has been shown to target downstream factors involved in cell cycle progression [110]. Also, MNK1 inhibitors, such as cercosporamide and eFT508, inhibits eIF4E phosphorylation and suppress tumor progression/metastasis in the xenograft and genetically engineered mouse models [111, 112]. One of the most common approaches is to combine MNKs inhibitors with mTOR inhibitors, due to the mutuality of these two pathways [113]. For validation of this approach a recent study demonstrated extended survival using mTOR inhibitor (rapamycin) in combination with tomivosertib in an APC KRAS colorectal cancer model [114]. Similarly, Fan *et a*l. found in hematological malignancies that mTOR deletion led to increased protein synthesis through MNKs, which may explain the resistance of cancer cells to mTOR inhibitors and provide importance of combination with MNK inhibitors and found resistant cancer cells sensitivity against the MNK inhibitor, CGP57380 [115]. Several clinical trials are ongoing to evaluate the anti-tumor efficacy and safety of MNK inhibitors, often in combinations, against varied cancers (Table 3). In particular, tomivosertib is currently in a Phase II clinical trial NCT03616834) to treat NSCLC patients and evaluate safety, tolerability, antitumor activity, and pharmacokinetics (NCT04622007).

**Table 3:** Development of single or combination therapy approaches for targeting MNK signaling pathway to treat various cancers

ClinicalTrials.gov identifier	Name of inhibitor	Conditions	Phase	Results/ Status
NCT03690141	Tomivosertib (eFT508)	Castrate-resistant Prostate Cancer (CRPC)	Phase II	Pharmacokinetics (PK), Dose toleration/ Completed
NCT02937675	Tomivosertib	Hematological Malignancies	Phase I and II	Dose-Escalation/ Terminated
NCT03616834	Tomivosertib + PD-1/PD-L1 Inhibitor	Solid Tumors	Phase II	Safety, dose toleration/ Completed
NCT03258398	eFT508 + Avelumab	Microsatellite Stable Relapsed or Refractory Colorectal Cancer	Phase II	Safety, dose toleration/ Recruiting/ Completed
NCT02605083	Tomivosertib	Advanced Solid Tumors	Phase I and II	Dose-Tolreation/ Terminated
NCT03318562	Tomivosertib	Advanced Triple Negative Breast Cancer and Hepatocellular Carcinoma	Phase II	Pharmacodynamic (PD) evaluation/ Terminated
NCT04261218	Tomivosertib + Paclitaxel	Advanced Breast Cancer	Phase I	PK-PD and Safety/Completed
NCT04622007	Tomivosertib + Pembrolizumab	Subjects With PD-L1 Positive NSCLC	Phase II	Progression free survival/ Recruiting
NCT03125239	Merestinib + LY2874455	Relapsed or Refractory Acute Myeloid Leukemia	Phase I	Dose toleration/ Recruiting
NCT03027284	Merestinib (LY2801653)	Advanced or Metastatic Cancer	Phase I	Dose toleration/ Recruiting/ Completed
NCT03292536	Merestinib	Bone Metastases, Breast Cancer	Phase I	Dose escalation/ Terminated
NCT02920996	Merestinib	Carcinoma, Non-Small-Cell Lung, Solid Tumor	Phase II	Overall Response rate (ORR) and Overall Survival (OS)/ Active not Recruiting
NCT02711553	Merestinib, Ramucirumab, Cisplatin, Gemcitabine	Biliary Tract Cancer, Metastatic Cancer, Advanced Cancer	Phase II	Progression Free Survival (PFS) and OS/Active
NCT02745769	Ramucirumab, Merestinib, Abemaciclib	Advanced Cancer, Colorectal Cancer, Mantle Cell Lymphoma	Phase I	Dose toleration/ Recruiting/ Completed
NCT02791334	LY3300054 Ramucirumab Abemaciclib Merestinib LY3321367	Solid Tumor Microsatellite Instability-High (MSI-H) Solid Tumors Cutaneous Melanoma Pancreatic Cancer Breast Cancer (HR + HER2 −)	Phase I	Safety and tolerability/ Active, not recruiting
NCT03414450	ETC-1907206 Dasatinib	Ph + Acute Lymphoblastic Leukemia (Ph + ALL) Ph- Acute Lymphoblastic Leukemia (Ph-ALL) Chronic Myeloid Leukemia Accelerated Phase (CML-AP, Ph +) Chronic Myeloid Leukemia Blast Crisis (CML-BC, Ph +)	Phase I	Safety and tolerability/ Withdrawn
NCT05462236	AUM001 Pembrolizumab Irinotecan	Metastatic Colorectal Cancer	Phase II	Safety and tolerability/ Recruiting
NCT02439346	BAY1143269, Docetaxel	Medical Oncology	Phase I	Dose toleration/ Terminated

### Targeting AMPK

AMPK plays a key role in several cancers by regulating various signaling pathways including mTOR. AMPK regulate cellular energy level and inhibiting it may disrupt cancer cell growth and metabolism. BAY-3827 is a specific inhibitor of AMPK. It has been investigated in preclinical studies as a potential cancer therapeutic due to its ability to inhibit AMPK, which plays a role in cellular energy regulation and metabolism. More recently, AMPK inhibitors BAY-3827 and SBI-0206965 were found to be efficient in inhibiting the proliferation of prostate cancer cell lines [116]. BAY-3827 inhibited human AMPK with a surprisingly low IC_50_ of 1.4 nM, while SBI-0206965 showed a similar potency [117]. BAY-3827 is now the inhibitor of choice for cell studies because of its impressive potency and limited off-target effects, even though its low bioavailability may limit its use in vivo [118]. However, like any potential cancer treatment, the efficacy and safety of AMPK inhibitors need to be carefully evaluated through clinical trials.

### Targeting alternatives of MYC

MYC stabilization is not a well-established aspect of MYC regulation, making it a topic of ongoing research in cancer biology. Studies have shown that indirect inhibition of MYC through targeting binding proteins and cofactors that can promote its stabilization and tumorigenicity have emerged as an alternative approach. We have illustrated the multiple pharmacological approaches to indirectly target MYC at distinct levels in Table 4. Aurora kinases are a family of serine/threonine kinases involved in cell division and implicated in MYC-amplified cancers. Aurora kinases A, B, and C are the key cell cycle progression regulators, especially in processes like mitosis and cytokinesis. Aurora kinase A causes tumorigenesis via communication with MYC [119, 120]. Aurora kinase A influences the cell cycle by making complexes with N-MYC and protecting them from FBW7-mediated proteasomal degradation [121]. The aurora kinase A inhibitors MLN8054 and MLN8327 unsettled the MYC-Aurora kinase A complex, leading to N-MYC destabilization and tumor deterioration in N-MYC amplified neuroblastoma [122]. Aurora kinases do not typically stabilize the C-MYC, but MLN8237 stimulated C-MYC degradation in p53 mutant hepatocellular carcinoma [123]. This data indicated that Aurora kinase A inhibitors could be possible therapeutics for treating MYC-amplified cancer and possibly interrupt cell division in MYC-amplified medulloblastoma. Another polo-like kinase (PLK) family is involved in the regulation of various cell cycle processes, including mitosis, cytokinesis, and DNA damage responses. Polo-like kinases, especially PLK1, have been shown to control essential biological processes in N-MYC amplified neuroblastoma and small cell lung carcinoma [124]. PLK1 inhibitors preferentially induce apoptosis of MYC-overexpressing tumor cells [125].

**Table 4:** Possible strategies for direct and indirect inhibition of MYC protein by small molecule to treat MYC-amplified cancer

MYC associated targets	Function in response to MYC stabilization	Inhibitors/inducers	References
BRD4	BRD4 can bind to acetylated lysine on the histones, and this interaction facilitate the recruitment and stabilization of MYC at specific loci	JQ1, OTX015, GSK2820151, ZEN-3694 CPI-0610, GSK925762, INCB057643	[86, 91, 101]
HDAC	HDAC regulates gene transcription by deacetylation, that can affect the turnover of MYC, influencing its level within the cell	CUDC-907, Panobinostat, Varinostat	[91, 141, 142]
HUWE1	HUWE1 can ubiquitinate MYC, but rather than it for degradation, this ubiquitination can lead to MYC stabilization. Ubiquitinated MYC might undergo other modifications that contribute to its stability and activity in the cell	BI8622, BI8626	[143–145]
MIZ1	MIZ1 may contribute to MYC stabilization by interfering with interaction between MYC and ubiquitin ligases that target MYC for degradation	BI8622, BI8626	[23, 146, 147]
PP2A	PP2A plays a role in stabilizing MYC through its involvement in post translational modification. PP2A can dephosphorylate MYC influencing its stability and activity	DT-061, FTY720, OP449, Perphenazine, LB-100	[148–150]
AURKA	AURKA has been linked to the stabilization of MYC through phosphorylation events. the specific mechanism may involve phosphorylation of MYC at certain sites, which can influence MYC stability and activity	MLN8237, CD532	[123, 151]
PLK1	PLk1 plays role in the regulation of MYC or MYC associated pathways that may involve complex interactions within the cellular signaling network	B16727	[125, 152]
FBXW7	In normal cellular process, FBXW7 helps regulate MYC levels by promoting its ubiquitination subsequent degradation. In this context, the loss of function contributes to increase MYC stability and activity	Oridonin	[153, 154]
MYC-MAX complex	MYC-MAX complex itself does not stabilize MYC it plays a central role in modulating the transcriptional activity of MYC. Together they act as transcription factor that regulate the expression of various gene involve in cell growth and proliferation	10058-F4, 10074-G5, Mycro3, KJ-pyr-9, sAJM589, MYCMi-6, MYCi975, Omomyc, KSI-3716, KI-MS2-008, NSC13728	[155–157]
USP7	USP7 plays role to stabilize MYC through its deubiquitinating activity	P22077, XL177A, GNE-6640, GNE6776, FT671	[158, 159]
PIN1	PIN1 plays a role in modulating the stability of and activity of various proteins including MYC, through its isomerase activity	Juglone, KPT6566, ATRA, BJP-06-005-3	[160, 161]
PRMT5	PRMT5 has been known to methylate arginine residues, leading to alteration MYC function. PRMT5 mediated to increase methylation has been linked to increased stability and transcriptional activity of MYC	JNJ-64619178, PF06939999, EPZ015666, GSK3326595, AMG 193, PRT543, PRT811, TNG908, MRTX1719, LLY 283, Compound1a, CMP5, GSK591, PRT382, JBI-778, SH3765, SCR6920	[162, 163]
CDK7	CDK7, which has kinase and cyclin-dependent kinase-activating kinase (CAK) activities that phosphorylate the C-terminal domain of RNA Pol II	THZ1	[164]
CDK9	CDK9, which has kinase and cyclin-dependent kinase-activating kinase (CAK) activities that phosphorylate the C-terminal domain of RNA Pol II and control several biological processes, including development differentiation and cell fate response	PC585, AZ5576	[165, 166]
JMJD6	JMJD6, has been implicated in MYC stabilization in certain cancers. It interacts with MYC and demethylates it, preventing proteasomal degradation. The demethylation activity of JMJD6 contributes to increased MYC stability, leading to sustained oncogenic signaling in MYC -associated cancer	WL12, SKLB325, J2	[167–169]

*HDAC* histone deacetylases, *BRD4* Bromodomain-containing protein 4, *HUWE1* HECT, UBA, and WWE domain-containing 1, *MIZ1* Myc-interacting zinc finger protein 1, *PP2A* protein phosphatase 2A, *AURKA* aurora kinase A, *PLK1* polo-like kinase 1, *FBXW7* F-box and WD repeat domain-containing 7, *USP7* ubiquitin specific protease 7 *PIN1* peptidyl-prolyl cis–trans isomerase NIMA-interacting 1, *PRMT5* protein arginine methyl transferase 5, *RNA Pol II* RNA polymerase II, *JMHD6* jumonji domain containing 6

It is important to note that the role of mTOR signaling in medulloblastoma can vary between individual cases and molecular subgroups. Therefore, treatment strategies may need to be tailored to the tumor's specific characteristics. Clinical trials have been conducted to evaluate the use of mTOR inhibitors, like rapamycin and its analogs, in treating medulloblastoma. These trials aim to assess the safety and effectiveness of the mTOR inhibitor in this specific context, and it is under investigation in several clinical studies for the treatment of pediatric tumors and other malignancies (Tables 1 and 2). It is important to note that mTOR inhibitors are not a one-size-fits-all solution, and their effectiveness can vary depending on the cancer's specific type and genetic characteristics. Additionally, resistance to mTOR inhibitors can develop over time, requiring ongoing research into novel strategies for targeting this pathway in cancer therapy.

### Targeting MYC-driven metabolism

MYC plays a central role in metabolic reprogramming by promoting an anabolic state in cancer cells [66]. Targeting such MYC-driven metabolic program using metabolic inhibitors could be one of the promising startegies for MYC-driven medulloblastoma. Particularly, in Group 3 medulloblastoma, MYC-driven metabolic alterations support rapid cell division and survival under stress. By inhibiting key metabolic pathways such as glycolysis, glutamine metabolism, and oxidative phosphorylation, the tumor’s energy production and biosynthetic processes can be interrupted, restricting its proliferation and survival [66, 126]. However, the complexity of metabolic programs in cancer cells and the potential for adaptive resistance require the development of combination therapies that target MYC-driven metabolism alongside other cellular pathways such as compensatory signaling pathways and DNA repair. Investigation on selective metabolic inhibitors and personalized treatment strategies will be crucial for overcoming resistance and improving outcomes in Group 3 (MYC-driven) medulloblastoma.

## Role(s) of MYC-mTOR signaling in chemoradition resistance

Resistance to chemoradiation therapy is a major challenge in treating Group 3 medulloblastoma. Both MYC and mTOR pathways have been implicated in this resistance [127, 128]. MYC can contribute to chemoradiation resistance by its control on cell cycle (cyclins and cyclin-dependent kinases) regulation, inhibition of apoptosis (anti-apoptotic factors; Bcl-2), metabolic reprogramming (glycolysis and oxidative phosphorylation), and DNA damage response [129, 130]. MTOR can contribute to chemoradiation resistance by its direct regulation of protein synthesis pathway (translation through 4EBP1/eIF4E), autophagy, and metabolism (nutrient uptake and processing) [129, 131]. The interaction between MYC and mTOR signaling pathways can create a robust network of resistance to therapy in medulloblastoma. MYC's promotion of cell cycle progression, apoptosis inhibition, and metabolic reprogramming synergizes with mTOR’s regulation of protein synthesis, cell survival, and autophagy. Therefore, targeting these pathways represents a promising strategy to overcome chemoradiation-resistance and improve treatment outcomes for patients with this challenging cancer.

## Possible resistance mechanisms of the targeting MYC-mTOR

Targeting MYC and mTOR signaling in medulloblastoma presents a promising approach to overcoming chemoradiation resistance, but several mechanisms of resistance could emerge in response to these treatments [129, 132] These mechanisms could either diminish the therapeutic effects of inhibitors targeting MYC and mTOR, or enable tumor cells to bypass the targeted pathways, thereby contributing to tumor persistence and recurrence [133]. Resistance to therapies targeting MYC and mTOR can arise through multiple mechanisms, including compensatory activation of alternative pathways (PI3K/AKT, MAPK/ERK), feedback loops (MYC-mTOR signaling feedback), tumor heterogeneity (clonal evolution), alterations in the tumor microenvironment (metabolic or hypoxic), and drug resistance through ABC Transporters (P-glycoproteins) [134–136]. Developing combination therapies that target these resistance mechanisms holds promise for overcoming treatment resistance and improving patient outcomes.

## Future perspective and conclusion

**Fig. 4:**
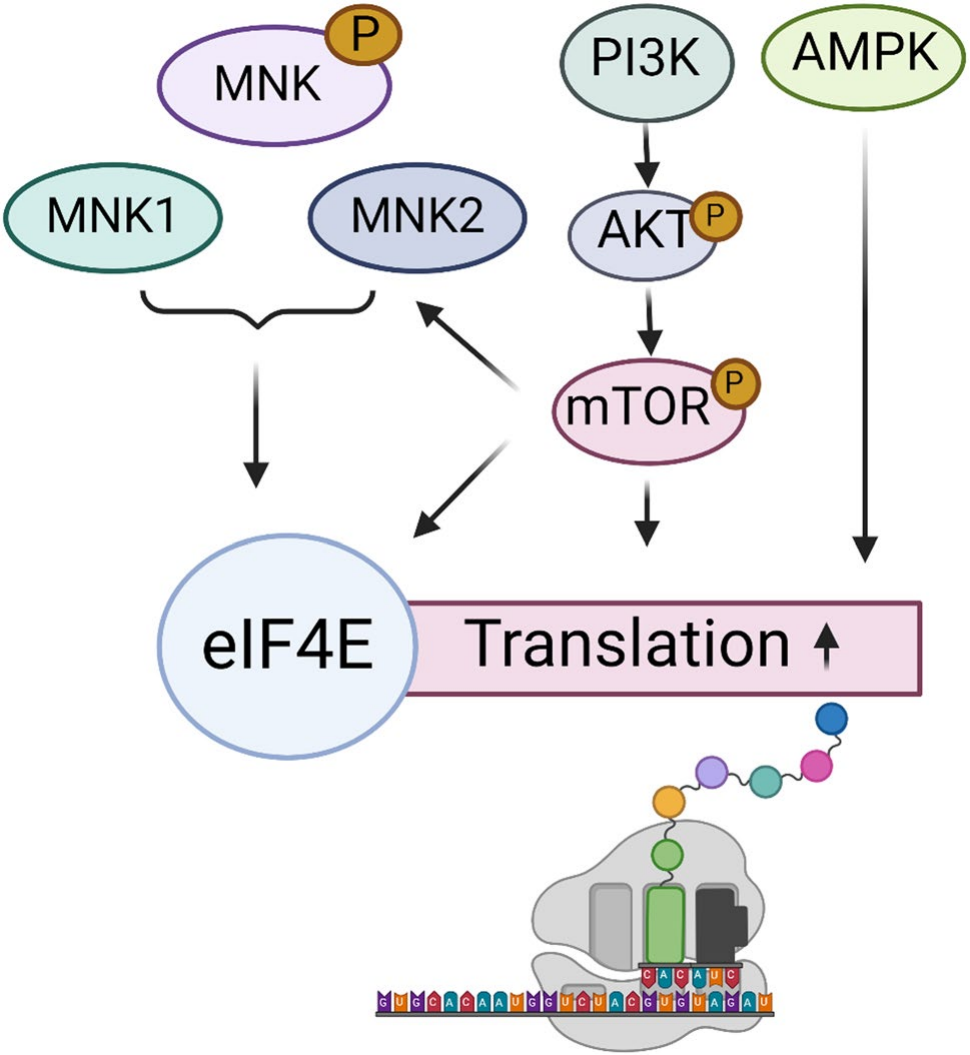
Other alternative strategies to target protein synthesis pathway in MYC-driven medulloblastoma. Activation of MNK, PI3K/AKT, and AMPK signaling pathways can lead to increased protein synthesis and tumorigenesis

The mTOR pathway plays one of the most prominent roles in tumor progression. It is linked with several pathways, and it factors into inhibition resistance, remarkably in highly resistant tumors such as MYC-driven medulloblastoma. Despite intensive multimodal therapy, the prognosis for Group 3 medulloblastoma patients with MYC-amplification remains extremely poor, and direct targeting of MYC has not yet been accomplished, but innovative approaches remain to be worked out towards realizing this goal. Whether via direct or indirect targeting of MYC, it is crucial to target MYC-associated pathways. However, despite substantial efforts, targeting MYC with clinical-grade small molecules still represents an intractable challenge, particularly when targeting MYC at the protein level. mTOR inhibitors are clinically available, as mentioned Tables 1 and 2. Recently evolving compounds that control or inhibit the mTOR signaling and its associated mechanisms, with possible utility for the treatment of various type of cancer including medulloblastoma, are summarized in Table 5. Targeting protein synthesis pathways in MYC-amplified medulloblastoma through mTOR inhibitors by combination therapy requires identifying complementary agents that can enhance therapeutic outcomes and overcome potential resistance mechanisms when combined with mTOR inhibitors. Such strategies may be multi-pronged, targeting various translation machinery components or exploiting vulnerabilities in MYC-amplified tumors (Figs. 3 and 4). By understanding the complex interplay of the signaling pathways, scientists hope to design more effective and personalized treatment regimens, ultimately improving the prognosis for individuals with MYC-amplified medulloblastoma. Realistically, a single drug approach is not reasonable for most cancer treatment and drug resistance is a most frequent challenge, therefore combination is necessary to utilized. Combining protein translation inhibitors (mTOR, MNK and AMPK), with MYC inhibitors may lead to a more comprehensive disruption of the pathways driving protein synthesis, potentially increasing the effectiveness of the treatments compared to single-agent therapies. MYC-amplified medulloblastoma often exhibits diverse genetic alterations contributing to treatment resistance. Combination therapy offers a strategy to overcome or mitigate resistance mechanisms, improving the chance of a positive clinical response. Optimizing the combination of mTOR and MYC-associated inhibitors has the potential to achieve therapeutic efficacy with lower doses of each drug, reducing the risk of adverse side effects and improving the overall tolerability of the treatments. Clinical trials are vital to evaluate the safety and efficacy of the combination therapies. Positive results from such clinical trials would validate the clinical relevance of this approach, leading to its potential integration into standard treatment protocols and holding promise in addressing the clinical challenges associated with MYC-amplified medulloblastoma.

**Table 5:** Preclinically evaluated compounds and reagents that have shown potential to inhibit mTOR signaling and protein synthesis

Test compounds	Mechanism	In vitro and in vivo models	References
MP1	Dual inhibitor of mTOR and MYC	HDMB-03 cell lines, HDMB-03 Xenograft Mice model	[170]
DL001	Inhibition of mTOR	PC cells, Mice embryonic fibroblast, C57BL/6 J mice	[171]
3HOI-BA-01	Inhibition of mTOR kinase and tumor growth	Non-small cell lungs cancer, mice,	[172]
RMC-4627	Inhibition of 4E-P1 phosphorylation, inhibition of cancer cell progression, viability, and survival	Acute lymphoblastic leukemia B cells, RMC-4627 cells, BCR-ABL cells	[173]
DHM25	Covalent inhibitor of mTOR and interfere AKT phosphorylation	Triple negative breast cancer cells	[174]
W922	Inhibition of cancer cells viability, enhance apoptosis and cell cycle arrest in G0-G1 phase	HCT116, MCF-7 and A549 cells, Mice xenograft models	[175]
Pf5212384	Inhibition of PI3K/mTOR Inhibition of NF-kB, AP-1 and IL8 Inhibition of cell proliferation and enhance apoptosis	14 HNSCC cell lines	[176]
JR-AB2-011	Inhibition of cancer cell growth and enhance the blood brain passage	LLC-PK1, LLC-mdr1a and LLC-MDR1 cells WT and KO mice with gliomas	[177]
GSK615	Inhibition of PI3K-AKT-mTOR, Inhibits the growth of gastric cancer cell and enhance apoptosis	Gastric cancer cells, Nude mice xenograft models	[178]
GDC-0084	Inhibitory effect on mTOR, Inhibition of cell proliferation and enhance apoptosis	MCF10A cells, brain metastasis xenograft mouse models	[179]
PQR309	Dual inhibition of PI3K/mTOR, Inhibition of cell proliferation and enhance apoptosis	GBM U87 cells, PC3 xenograft model in nude rats	[180]
MCX83	Dual inhibition of PI3K/mTOR	Cancer cell lines	[181]
Torin 1	mTOR inhibitor	Cancer cell lines,	[182]
PP242	mTOR inhibitor, reduces expression of p-S6K1 and the partially reduced phosphorylation of 4E-BP1	colorectal carcinoma (CRC) cell lines	[183]
PP30	PP30 inhibits mTORC1 and mTORC2 in an ATP-competitive manner and had greater impacts on cell cycle, cell growth and proliferation, and cap-dependent translation rather than the prototype inhibitor rapamycin	Cancer cell lines,	[184]
WYE-354	WYE-354 inhibits both mTORC1 and mTORC2. WYE-354 induces autophagy activation	Caco-2 Cell line	[185]
WYE-132	WYE-354 blocks mTORC1/2 activation and inhibited expression of mTOR-regulated genes (cyclin D1 and hypoxia-inducible factor 1α)	Ovarian cancer cell line	[186]
OSI-027	OSI-027 inhibits phosphorylation of the mTORC1 substrates 4E-BP1 and S6K1 as well as the mTORC2 substrate AKT in diverse cancer models in vitro and in vivo	Cancer cell lines, Female CD-1 Mice	[187]

The blood–brain barrier (BBB), involving multidrug-resistant membrane proteins like P-glycoprotein (P-gp), breast cancer resistance protein (BCRP), poses a challenge in delivering drugs to the brain. BBB plays a crucial role in limiting the entry of substances, including drugs, into the brain [137]. Insufficient drug transport into the brain leads to diminished therapeutic effects and aggravated organ toxicity side effects due to the deposition of the drug in other organs and tissues [138]. In the context of treating medulloblastoma, especially when targeting the mTOR pathway with inhibitor drug, the importance of understanding and overcoming the BBB is significant. Many mTOR inhibitors are substrates for efflux pumps like P-gp and BCRP that reduce the efficacy of the drugs. Some mTOR inhibitors like everolimus and temsirolimus are the substrate of Pgp and BCRP. These efflux pumps can influence the absorption, distribution, and elimination of the mTOR inhibitors and other combinations, impacting their pharmacokinetic properties [139]. To ensure optimal efficacy, potential drug interactions should be considered when using mTOR inhibitors in a clinical setting. Ensuring effective penetration of BBB by all components of the combination is critical. For example, a combination of ribociclib with BET-bromodomain and PI3K/mTOR inhibitors were used for the treatment of medulloblastoma [99]. Brain penetration was variable among all existing inhibitors. Paxalisib (mTOR inhibitor) was specially designed to cross the BBB and showed an excellent brain-to-plasma ratio [140]. JQ1 (a BET inhibitor) failed to show efficacy due to high clearance and insufficient brain penetration. Another preclinical study has shown the synergistic effect of JQ1 with BEZ235 (PI3K/mTOR inhibitor) and JQ1 with temsirolimus on a medulloblastoma spheroid model and a MYC-driven medulloblastoma xenograft [39]. This combination remains to be conducted at the clinical level.

Researchers are exploring strategies to enhance drug delivery across the BBB, such as nanoparticle-based drug delivery systems or temporary disruption of the barriers. Overcoming the challenge of BBB is crucial to ensure that mTOR inhibitors and combination inhibitors associated with MYC translation effectively reach medulloblastoma cells in the brain, maximizing the therapeutic impact and improving therapeutic outcomes for patients. Advances in addressing BBB issues could pave the way for more successful treatment for brain tumors like medulloblastoma.

Combination of multiple therapies may raise the risk of drug toxicities and side effects, affecting patients’ quality of life and restricting the tolerability of the treatments. Determining optimal doses of each component of the combination can be challenging, as interaction between drugs may affect their pharmacokinetics and pharmacodynamics.

Addressing these hurdles requires a collaborative effort among researchers, clinicians, and pharmaceutical companies. Rigorous preclinical and clinical studies and advancements in drug development and delivery technology are essential for overcoming these challenges and realizing the potential benefits of combination therapy to target protein translation for Group 3 MYC-amplified medulloblastoma.

## Data Availability

No datasets were generated or analysed during the current study.
